# Notes and News

**Published:** 1894-12-08

**Authors:** 


### Notes and News.

Collected and Communicated.

Mr. F. W. Howell, steward of the *Dreadnought* Seamen's Hospital, Greenwich, has been appointed Secretary and Manager of the York County Hospital *vice* Mr. C. E. Pinfold, who has been called to the Bar, and intends practising in London.

The generous offer of £1,000 made by the Goldsmiths' Company in aid of research work regarding the utility of antitoxins in the treatment of diphtheria will help to solve the very serious questions which have arisen as to the supply of serum for the use of the public.

The Victoria Hospital for Consumption and Diseases of the Chest, Edinburgh, was opened on November 22nd by Lord Stormouth-Darling. The rapid increase in the number of patients necessitated removal to larger premises. Craig Leith House, a commodious mansion, situated in a wooded park, has been adapted as a hospital. Accommodation is now ready for fifteen patients.

We have received the following appeal on behalf of the North-Eastern Hospital for Children from the chairman of committee, vice-president, and the Bishop Suffragan for East London. The crowded inhabitants of the north-east and east of London depend very largely on the North-Eastern Hospital for Children, Hackney Road, Shoreditch, for the relief of their children in the all too frequent hours of sickness and of accident, but this institution, founded twenty-seven years ago, is now in such distress as to be in danger of having to curtail, if not to altogether abandon, its work of treating some 17,000 children annually. The committee have now in hand £94 only, while accounts for the past three months, amounting to £818, are still unpaid, and the Christmas bills, amounting to another £600, will soon be due. Add to this that there is already a debt (on mortgage at 4 per cent.) of £4,500, resulting from the deficits of past years, and the position of the hospital can be realised. We claim that the maintenance of this hospital is a matter of public importance, and we therefore appeal to each one of your readers to subscribe some amount, no matter how small, and thus to at once dispel its difficulties. We would ask that contributions be sent to the Secretary at the Hospital, and that cheques, &c., be crossed Barclay, Bevan, and Co.

A new wing has been opened at the Women's Free Hospital, Southampton. It consists of out-patients accommodation and doctors'-rooms.

The annual dinner of past and present students of the National Dental Hospital and College was held on Friday last, Sir Walter B. Foster, M.P., presiding.

A gentleman who desires to be anonymous has promised to subscribe £25 to the North Eastern Hospital for Children, provided nine others will each give a like sum before December 14th.

The new floating cholera hospital in the Tees is now ready. The building consists of two ward blocks with an administrative block between them. Behind these are laundry, mortuary, and destructor. The float of the hospital consists of ten cylinders, 86½ ft. long by 6 in. diameter. The cost of the hospital has been about £10,000.

The Bishop of Southwark, acting for the Bishop of Rochester, who is indisposed, dedicated the chapel at the British Home for Incurables last week. The chapel is the gift of Miss Leicester, and has cost £1,800. A pleasing circumstance in connection with this event and the opening of the new home at Streatham last July is the presentation of a handsome silver goblet to the secretary, Mr. R. Gofton Salmond, from the in-patients as a mark of their esteem and affection for him.

We learn that the entire medical staff, having approved of the admission of lady medical students to the Dundee Royal Infirmary, the directors have given their sanction to the scheme. For the present the teaching of the two sexes will be combined, except in special cases. This is only a temporary arrangement, which, when the classes increase, will be subject to alteration. At present we are told that the whole number of students studying consists of four—two male and two female.

With reference to the evidence given at the inquest on John Round, at the St. Pancras Coroner's Court on November 30th, the Board of Management of the London Temperance Hospital desire to record the following facts: The patient applied at, and was admitted into the hospital on November 8th suffering from ulcers of the leg. He had also a festered finger, which might have arisen from a cut from his lathe, to which cause, however, neither he nor his wife attributed his illness. The case-notes show that he was in the hospital ten days, during which time the ulcers began to heal; he had no symptoms of any blood poisoning or fever, his temperature, according to the chart, being hardly above normal; his organs were apparently healthy, but he was somewhat emaciated, and of a morose disposition. On the evening of November 15th he became melancholic, refused his food, would not answer when spoken to, and was violent and noisy at night. Accordingly on November 16th his wife was sent for, informed of his mental condition, and asked whether she would take him home, or prefer that the relieving officer of the parish should be communicated with. She took some time to decide, but finally made up her mind that the latter course should be adopted. The patient was, therefore, removed under the care of the relieving officer. In the opinion of five medical men who saw the case it was one of acute melancholia arising in a debilitated subject, not one of delirium due to accidental cause or blood poisoning. Cases of the latter class are treated in the separate wards provided within the hospital for such purpose. The only alternatives open for the proper care and treatment of such a case were duly explained to the patient's wife.

